# Elevated ADH5 expression suggested better prognosis in kidney renal clear cell carcinoma (KIRC) and related to immunity through single-cell and bulk RNA-sequencing

**DOI:** 10.1186/s12894-024-01478-9

**Published:** 2024-04-10

**Authors:** Junhao Sun, Xinyu Zhang, Fan Wu, Bingye Zhu, Huyang Xie

**Affiliations:** 1 Department of Urology, Affiliated Hospital of Nantong University, No.20 West Temple Road, Nantong, 226001 Jiangsu Province China; 2 Department of Urology, Renji Hospital, School of Medicine, Shanghai Jiao Tong University, Shanghai, 200127 China; 3 Department of Urology, Affiliated Nantong Hospital of Shanghai University (The Sixth People’s Hospital of Nantong), No. 881 Yonghe Road, Nantong, 226001 Jiangsu Province China

**Keywords:** Kidney renal clear cell carcinoma, Overall survival, ADH5, Immunity

## Abstract

**Background:**

Despite the rapid advances in modern medical technology, kidney renal clear cell carcinoma (KIRC) remains a challenging clinical problem in urology. Researchers urgently search for useful markers to break through the therapeutic conundrum due to its high lethality. Therefore, the study explores the value of ADH5 on overall survival (OS) and the immunology of KIRC.

**Methods:**

The gene expression matrix and clinical information on ADH5 in the TCGA database were validated using external databases and qRT-PCR. To confirm the correlation between ADH5 and KIRC prognosis, univariate/multivariate Cox regression analysis was used. We also explored the signaling pathways associated with ADH5 in KIRC and investigated its association with immunity.

**Results:**

The mRNA and protein levels showed an apparent downregulation of ADH5 in KIRC. Correlation analysis revealed that ADH5 was directly related to histological grade, clinical stage, and TMN stage (*p* < 0.05). Univariate and multivariate Cox regression analysis identified ADH5 as an independent factor affecting the prognosis of KIRC. Enrichment analysis looked into five ADH5-related signaling pathways. The results showed no correlation between ADH5 and TMB, TNB, and MSI. From an immunological perspective, ADH5 was found to be associated with the tumor microenvironment, immune cell infiltration, and immune checkpoints. Lower ADH5 expression was associated with greater responsiveness to immunotherapy. Single-cell sequencing revealed that ADH5 is highly expressed in immune cells.

**Conclusion:**

ADH5 could be a promising prognostic biomarker and a potential therapeutic target for KIRC. Besides, it was found that KIRC patients with low ADH5 expression were more sensitive to immunotherapy.

**Supplementary Information:**

The online version contains supplementary material available at 10.1186/s12894-024-01478-9.

## Introduction

The incidence of urinary tract malignant tumors increases with age [1]. Renal cell carcinoma (RCC) is one of the most lethal malignant tumors in the urinary system, and 75–80% of the pathological types are kidney renal clear cell carcinoma (KIRC) [2]. It has been reported that about 30% of patients with KIRC develop metastasis at the time of initial diagnosis [3]. Despite the rapid development of new methods to combat KIRC, it is not sensitive to radiotherapy and chemotherapy, and most patients will eventually develop drug resistance and relapse [4]. Due to the high morbidity and mortality rates of KIRC patients [5], it is imperative to identify new prognostic biomarkers and therapeutic targets. This will help to improve the prognosis and personalize the treatment of KIRC patients.

As a member of the alcohol dehydrogenase (ADH) family, ADH5 is a relevant player in inflammation, immune regulation, and cancer progression, as well as many other processes, and its dysregulation can upset cellular homeostasis and cause disease [6, 7]. Research has demonstrated that the downregulation of ADH5 is a feature of primary hepatocellular carcinoma, and its pharmacological inhibition by inducible nitric oxide synthase (iNOS) presents a promising treatment option for individuals with primary hepatocellular carcinoma [8, 9]. In addition to this, researchers have found that ADH5 acts as a player in cancers such as breast [10], ovarian [11], and prostate cancers [12].

With the rapid development of bioinformatics, it is possible to identify key genes associated with tumor prognosis and progression with bioinformatics analysis. Several potential KIRC biomarkers have now been obtained through TCGA data mining. These biomarkers were then further validated against other datasets, culminating in the discovery of the gene ADH5, which has not been studied in relation to KIRC before. Therefore, this paper focused on the effect of ADH5 on the prognosis of KIRC and discussed even further the connection between ADH5 and immunization and immunotherapy. It is hoped that our findings will hopefully provide promising therapeutic candidates for KIRC for future targeted therapies and improve patient prognosis.

## Materials and methods

### Data collection and processing

Gene expression matrices associated with ADH5 and clinical data from KIRC patients were extracted from The Cancer Genome Atlas (TCGA; http://cancergenome.nih.gov/) database, including 539 KIRC tumor samples and 72 non-malignant tumor tissue samples. Genetic profiles and clinical information were then extracted for further analysis. Cases were finally excluded if there were incomplete or missing data, such as age or overall survival (OS). The ADH5 gene matrix was further analyzed, as was the corresponding clinical information. R software (https://www.r-project.org/) was used for all data processing. Calculation of the different expression levels of ADH5 mRNA used by the “Limma” package in the R package. |log2 fold change (FC)|≥1 and an adjusted P value (FDR) < 0.05 were defined as truncation criteria.

### Identify differentially expressed associated genes and perform gene set enrichment analysis (GSEA)

To identify differentially expressed genes (DEGs), we analyzed the differential expression data for the ADH5 gene in tumors, and quasi-tumor non-tissues were analyzed using the ‘Limma’ package. In addition, we performed a gene set enrichment analysis (GSEA) to investigate the biological functions and signaling pathways associated with ADH5 [13].

### Validation of ADH5 protein expression in tissues

The UALCAN website (http://ualcan.path.uab.edu/analysis-prot.html) was used to analyze the CPTAC dataset and determine ADH5 expression in primary KIRC tissue and normal tissue. The expression of ADH5 protein in KIRC was validated immunohistochemically using the HPA online database (http://www.proteinatlas.org/).

### Quantitative real-time PCR (qRT-PCR)

ADH5 mRNA expression was detected in four KIRC cells and normal renal tubular epithelial cells. The reaction system consisted of 10µL of SYBR QPCR Master Mix (High Rox premix), 0.4µL of forward primer, 0.4µL of reverse primer, 4.2µL of ddH2 O, and 5µL of cDNA. Glycerol 3-phosphate Hyde Dehydrogenase (GAPDH) was employed for internal reference. The sequence of primers is as follows: ADH5: F: 5’-TGCTGCTGTGAACACTGCCAAG-3’ and R: 5’-CCATGATAACTGCCAATCCGACTCC-3’; GAPDH: F: 5’-CAGGAGGCATTGCTGATGAT-3’; R: 5’-GAAGGCTGGGGCTCATTT-3’. All data are expressed as mean ± standard deviation according to the 2^−ΔΔCt^ method. The software used for statistical analysis and plotting is GraphPad Prism 8 (GraphPad Software, San Diego, CA, USA). *P* < 0.05 suggests that the differences are statistically significant.

### Correlation of the prognosis of ADH5 and KIRC patients

Univariate/multifactorial Cox risk regression analysis was performed to investigate whether ADH5 was an independent influence on OS in KIRC cases based on a total of eight clinical factors (race, gender, age, grade, T, M, N, stage) and the corresponding KIRC patients in the TCGA database.

### An analysis of the correlation between ADH5 and microsatellite instability (MSI), tumor mutational load (TMB), tumor neoantigen load (TNB), and immunological evaluation

The results of this part of the analysis are visualized on the Sangerbox website (http://www.sangerbox.com/tool). In the single gene pan-cancer analysis module available on this website, the Pearson method was used for correlation analysis. The aim was to investigate the association between ADH5 expression and MSI, TMB, and TNB, with a threshold set at less than 0.05 [14, 15].

### Correlation of ADH5 in the immune infiltration and tumor microenvironment

The relevance between ADH5 and immune cell infiltration in KIRC was investigated by the TIMER analysis tool (https://cistrome.shinyapps.io/timer/). The ESTIMATE method utilizes existing gene expression profiles to estimate the degree of infiltration of stromal or immune cells into the tumor [16]. So, based on the ADH5 expression matrix, we executed the ESTIMATE algorithm to calculate the immune, stromal, and ESTIMATE scores. Sangerbox was also used to investigate the relationship between ADH5 and immune checkpoints and immune cells.

### Prediction of ADH5 for responsiveness to immunotherapy

The Tumor Immune Dysfunction and Exclusion Database (http://tide.dfci.harvard.edu) is used to infer the function of genes that regulate tumor immunity. It takes advantage of T-cell dysfunction and rejection to model immune escape from tumors and thus effectively forecast the effectiveness of immune checkpoint suppression therapy [17]. The TIDE algorithm was used to determine the potential of ADH5 to predict KIRC immunotherapy.

### Single-cell sequencing data sources and analysis

From the GEO database, we downloaded three datasets: GSE111360, GSE159115, and GSE139555. Both renal tumor and normal kidney tissue sequencing data are included in these datasets. From the GSE121636 dataset, single-cell sequencing of peripheral blood from tumor patients has been downloaded. We set the criteria for high-quality cells as > 500 genes/cell and < 15% mitochondrial genes. Principal Component Analysis (PCA) within the Seurat package in the R language is used to reduce dimensionality, followed by visualization via Unified Streaming Approximation and Projection (UMAP). The FindClusters function was used to identify major cell clusters and was annotated with a resolution value of 0.5. The above results are presented in the form of UMAP plots, speckle plots, and violin plots.

## Results

### Expression levels of ADH5 in KIRC

**Fig. 1:**
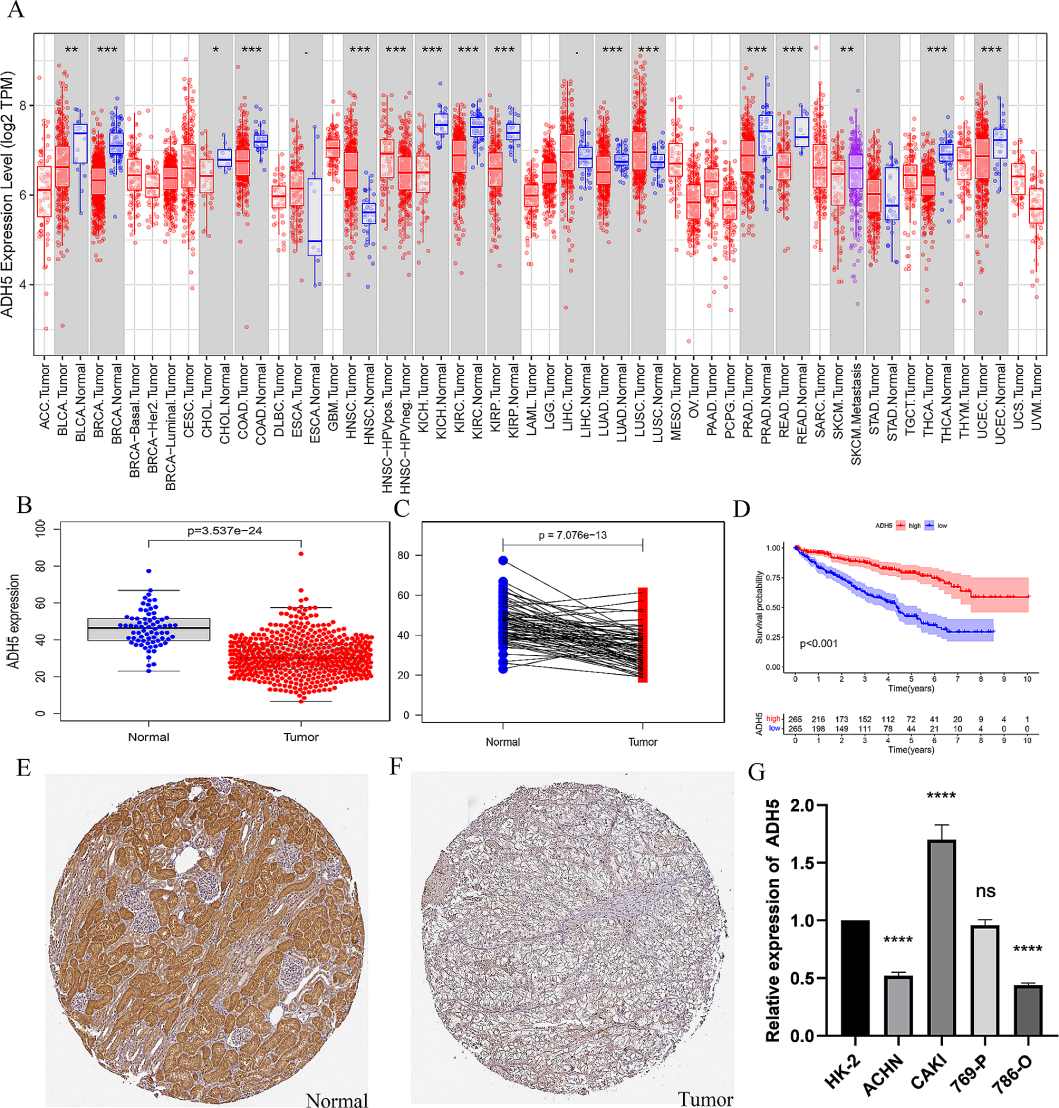
Expression of ADH5 in KIRC. (**A**) The expression of ADH5 in various cancers in the TCGA database; (**B**) ADH5 expression plots in normal kidney tissues and KIRC tissues; (**C**) ADH5 paired plots in normal kidney tissues and KIRC tissues; (**D**) Kaplan-Meier curves suggest an association between ADH5 expression and OS; (**E**-**F**) Immunohistochemical staining of ADH5 in the HPA database; (**G**) Expression levels of ADH5 in four types of renal cancer cells. *P < 0.05, **P < 0.01, ***P < 0.001

To investigate the differential expression of ADH5 in tumor and normal tissues, the mRNA expression levels of ADH5 in TCGA-PAN-cancer tissues were analyzed, and results were obtained as follows (Fig. 1A). Concurrently, the expression of ADH5 in tumor tissue was significantly lower compared to the adjacent normal tissue (*P* < 0.001, Fig. 1B). The same result was found by comparing the profiles (*P* < 0.001, Fig. 1C). KM survival curves demonstrated greater survival over time in KIRC patients with high ADH5 expression compared to those with low expression (Fig. 1D). Immunohistochemical staining from different patients in the HPA database (http://www.proteinstallas.org/) showed that ADH5 expression in tumor tissues of KIRC patients was significantly lower than that in normal renal tissues (Fig. 1E and F). Further PCR results suggested that ADH5 was down-regulated in ACHN and 786-O, two KIRC cells, compared to human normal renal tubular epithelial cells (HK-2). In addition, ADH5 showed upregulation in CAKI cells (Fig. 1G). In conclusion, combined with the above results, ADH5 may be an anti-oncogene with low expression in KIRC, and the increase of ADH5 indicates a better prognosis.

### Expression of total protein or phosphorylated protein expression levels of ADH5 in KIRC

**Fig. 2:**
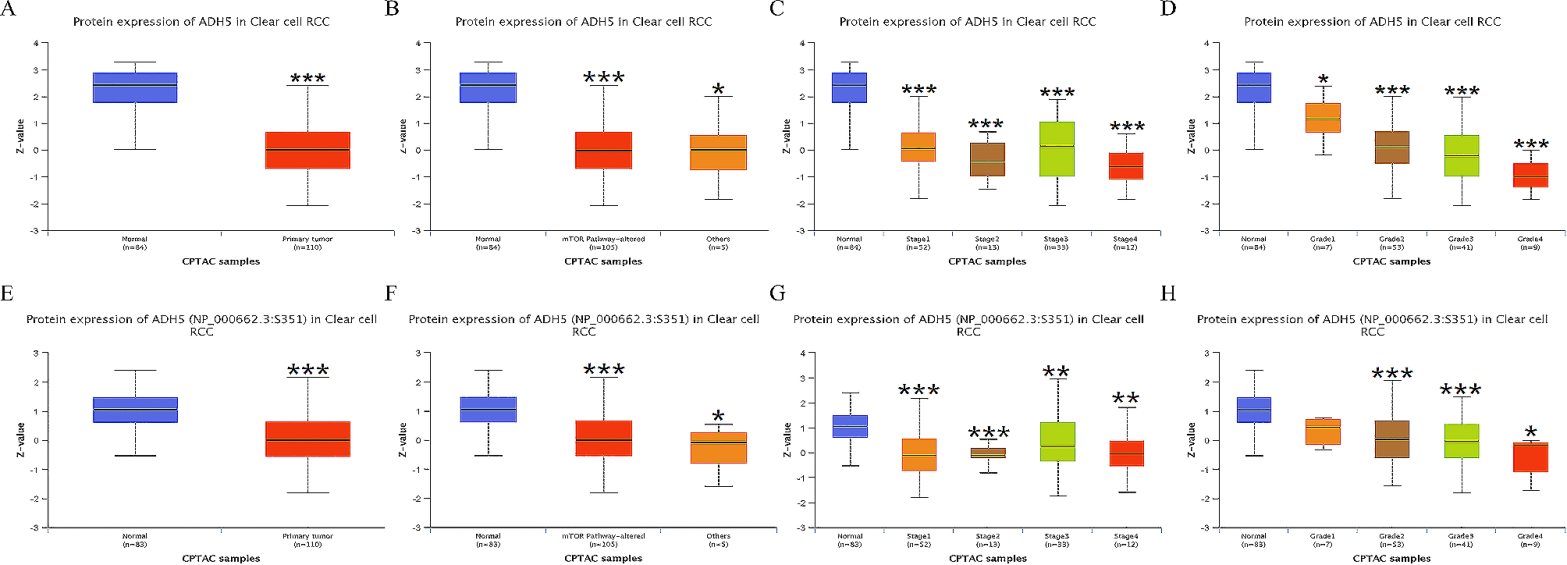
The expression and distribution of ADH5 protein in the CPTAC database. (**A**) Protein expression of ADH5 in natural kidney tissues and KIRC primary tumor tissues; (**B**) Protein expression of ADH5 in the normal group, mTOR pathway altered group, and other groups; (**C**) ADH5 protein expression in different stages; (**D**) ADH5 protein expression in different grades. (**E**) ADH5 phosphoprotein expression at the S351 locus in normal kidney tissues and KIRC primary tumor tissues; (**F**) ADH5 phosphoprotein expression at the S351 locus in the normal group, mTOR pathway altered group, and other groups; (**G**) Protein expression at the ADH5 phosphoprotein at the S351 locus in different stages; (**H**) Protein expression at the ADH5 phosphoprotein at the S351 locus in different grades. *P < 0.05, **P < 0.01, ***P < 0.001

The results of the CPTAC study of total ADH5 protein and phosphoprotein expression in KIRC via the UALCAN website showed that the expression of total ADH5 protein was lower in primary KIRC tumors than in normal kidney tissue (*P* < 0.001). ADH5 was expressed at the S351 phosphorylation site in tumor tissues with an altered mTOR pathway. The ADH5 phosphoprotein exhibited expression at the S351 phosphorylation site in both primary KIRC and tumors with dysregulated mTOR pathways, demonstrating significantly reduced levels compared to normal renal tissues. Additionally, the distribution of ADH5 protein expression at various grades or stages is clearly presented (Fig. 2A-H).

### ADH5 mRNA expression levels correlate with clinicopathological parameters

**Fig. 3:**
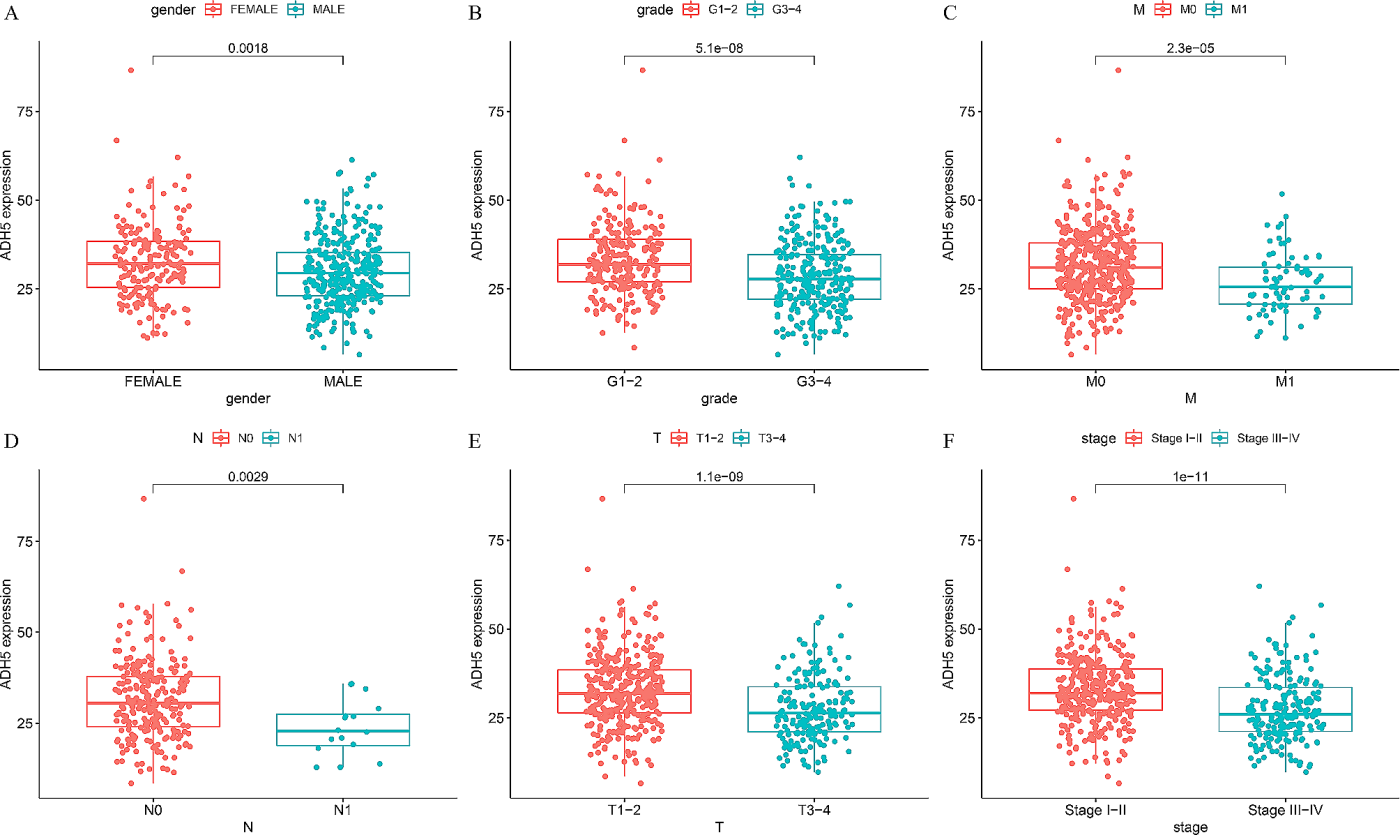
The relationship between ADH5 and (**A**) gender, (**B**) grade, (**C**) M, (**D**) N, (**E**) T, and (**F**) stage

Logistic regression analysis was performed on six clinical characteristics: gender, grade, stage, T, M, and N, in the clinical data of KIRC patients in the TCGA. The results showed a significant association between ADH5 expression and gender (*P* = 0.018), grade (*P* = 5.1E-08), stage (*P* = 1E-11), T stage (*P* = 1.1E-09), and M stage (*P* = 2.3E-05), as well as a significant correlation with N stage (*P* = 0.0029) (Fig. 3). From these results, we conclude that reduced ADH5 expression in KIRC patients correlates with tumor progression.

### The relationship between ADH5 and KIRC prognosis

**Fig. 4:**
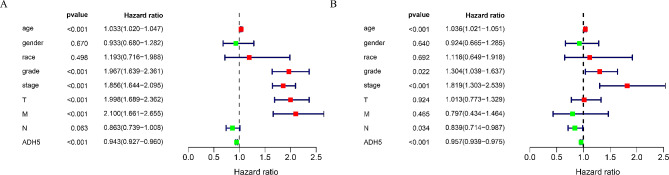
To verify whether ADH5 is an independent factor influencing the prognosis of KIRC. (**A**) univariate Cox regression analysis; (**B**) multivariate Cox regression analysis

The univariate Cox regression analysis demonstrated that age, grade, stage, T-stage, M-stage, and ADH5 expression were significantly correlated with OS (all *P* < 0.05; Fig. 4A; Table 1). The results of the multifactorial Cox regression analysis showed significant correlations between age, grade, stage, N stage, and ADH5 expression with OS in KIRC (all *P* < 0.05; Fig. 4B; Table 1). Based on these results, we concluded that ADH5 expression, age, stage, and grade may have an independent effect on the OS in KIRC patients.

**Table 1:** Univariate and multivariate analyses of ADH5 and clinicopathologic factors of overall survival in ccRCC

Factors	Univariate analysis				Multivariate analysis			
	HR	HR.95 L	HR.95 H	*p* value	HR	HR.95 L	HR.95 H	*p* value
**age**	1.033274	1.019678	1.047052	**1.28E-06**	1.035733	1.020882	1.050801	**1.89E-06**
**gender**	0.933298	0.679692	1.28153	0.669603	0.924393	0.665222	1.284538	0.63955
**race**	1.193075	0.71596	1.988138	0.498059	1.115822	0.649279	1.917602	0.691606
**grade**	1.966884	1.638836	2.360598	**3.70E-13**	1.303948	1.038568	1.63714	**0.02226**
**stage**	1.855626	1.643637	2.094956	**1.71E-23**	1.818668	1.302577	2.539238	**0.000444**
**T**	1.997582	1.689052	2.362469	**6.29E-16**	1.013337	0.772911	1.328553	0.923616
**M**	2.099647	1.660681	2.654644	**5.70E-10**	0.797177	0.434168	1.4637	0.464686
**N**	0.862971	0.73887	1.007916	0.062826	0.839073	0.713649	0.986541	**0.03367**
**ADH5**	0.943382	0.927019	0.960033	**6.63E-11**	0.956608	0.938989	0.974557	**2.91E-06**

Bold values means *P* < 0.05

### Identification of ADH5-related signaling pathways based on GSEA

**Fig. 5:**
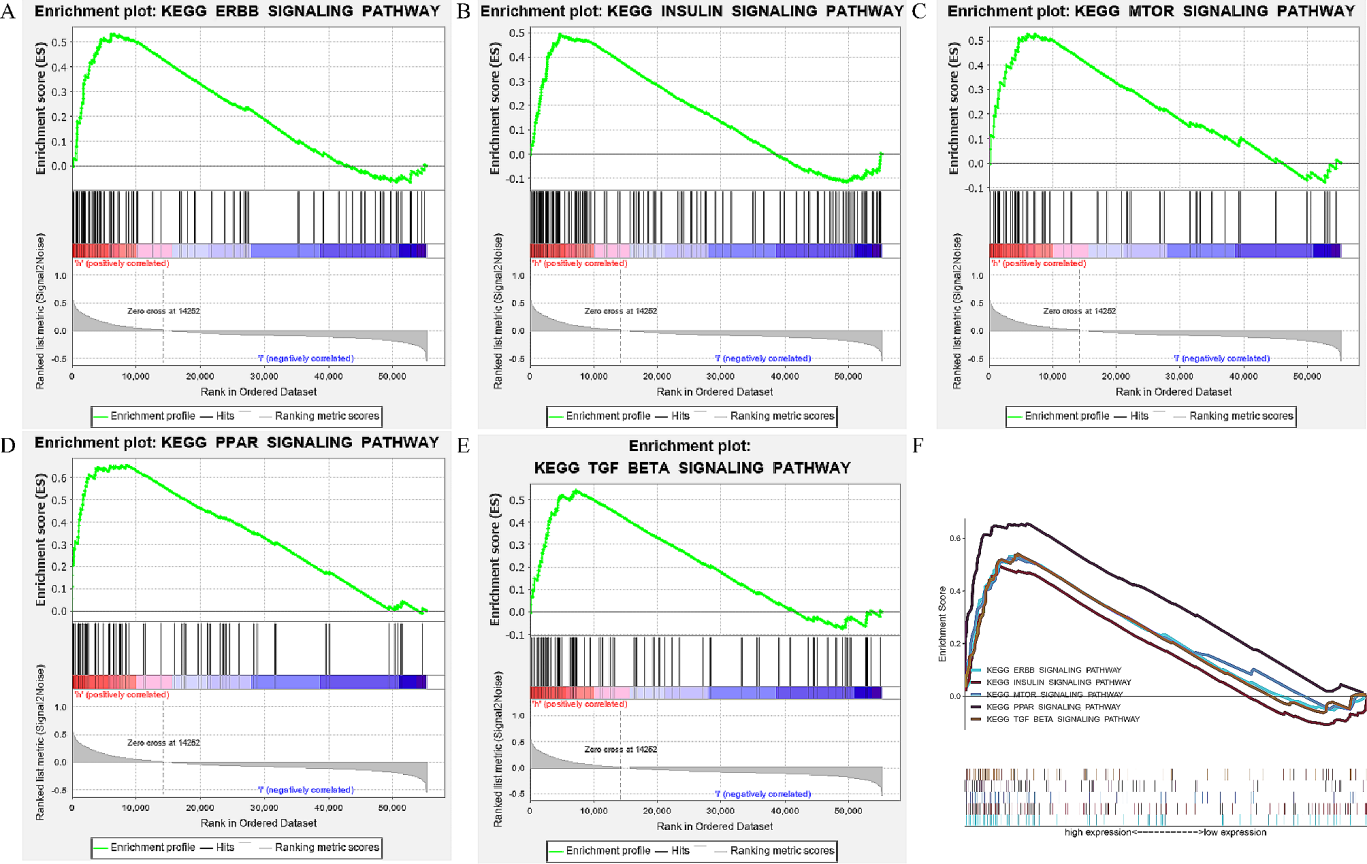
Gene set enrichment analysis (GSEA) enrichment map. (**A**) ERBB SIGNALING PATHWAY; (**B**) INSULIN SIGNALING PATHWAY; (**C**) MTOR SIGNALING PATHWAY; (**D**) PPAR SIGNALING PATHWAY; (**E**) TGF BETA SIGNALING PATHWAY

We used GSEA assays to identify signaling pathways associated with ADH5 between high ADH5 and low ADH5 expression matrices and determined five signaling pathways, including ErbB, Insulin, mTOR, and PPAR, in addition to TGF-β, by setting *P* values less than 0.05 and normalized enrichment scores greater than 1.5 (Fig. 5; Table 2). These results assist in further investigating the pathogenesis of KIRC.

**Table 2:** Gene set enrichment analysis (GSEA) of ADH5 in ccRCC

GeneSet name	NES	Nominal *p*-value	FDR q-value
ERBB SIGNALING PATHWAY	2.016981	0.001904762	0.015454717
INSULIN SIGNALING PATHWAY	2.0507483	0.001949318	0.013348792
MTOR SIGNALING PATHWAY	1.9145837	0.007575758	0.022305226
PPAR SIGNALING PATHWAY	2.1627238	0.002040816	0.00683311
TGF BETA SIGNALING PATHWAY	1.8403625	0.023904383	0.032773167

### Relationship between ADH5 and PPI, MSI, TMB, TNB in KIRC

**Fig. 6:**
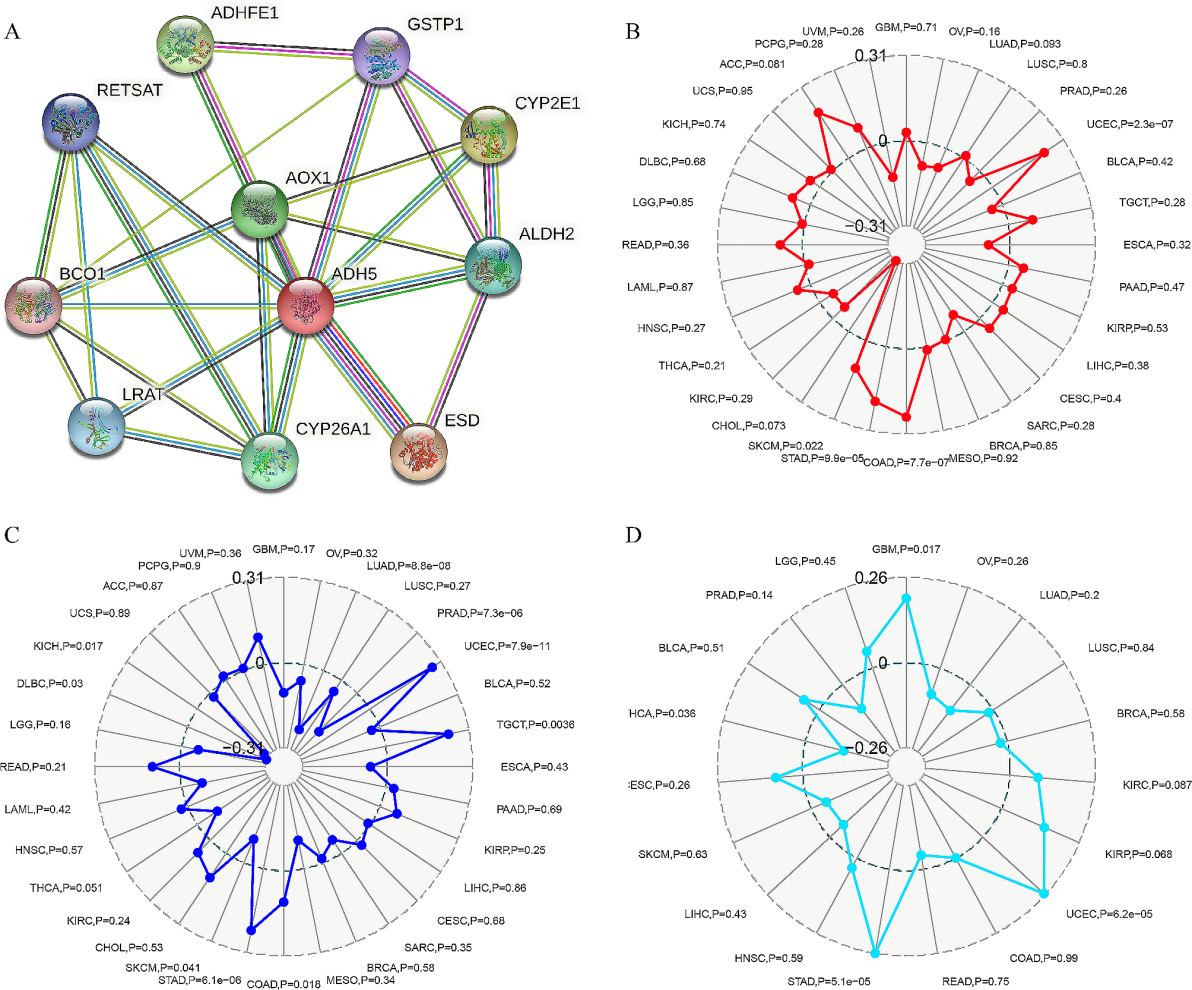
ADH5 about (**A**) PPI; (**B**) TMB; (**C**) MSI; (**D**) TNB

We constructed possible relationships between ADH5 and other KIRC genes, including those in 11 genes, by searching the online STRING database (https://string-db.org/) to explore potential functional interactions of ADH5 (RETSAT, BCO1, LRAT, CYP26A1, ADH5, AOX1 ADHFE1, GSTP1, CYP2E1, ALDH2, ESD, Fig. 6A) [18]. In parallel, we assessed the relationship of ADH5 expression with MSI, TMB, and TNB by Pearson’s method through the Sangerbox website, with the threshold set at *P* < 0.05. The results showed no significant correlation between ADH5 and KIRC-associated MSI, TMB, and TNB (Fig. 6B-D).

### Relationship of ADH5 to the KIRC tumor microenvironment, tumor immune infiltration, immune cell pathways, and immune checkpoint molecules

**Fig. 7:**
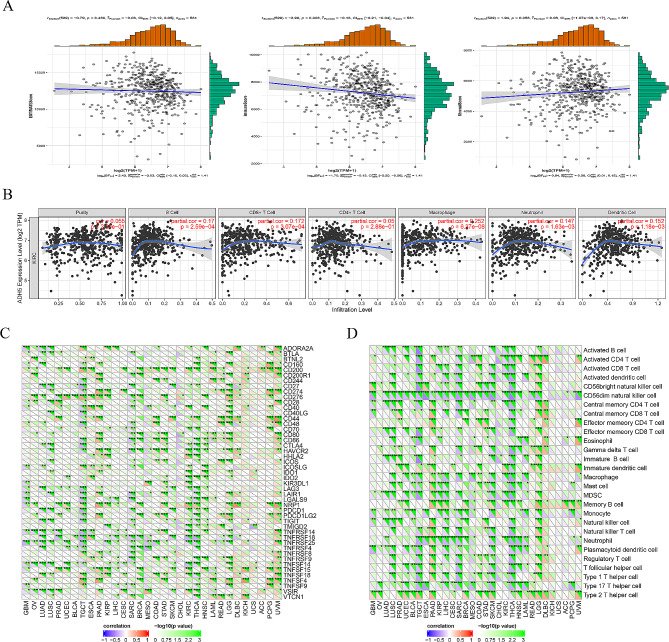
ADH5 about (**A**) tumor microenvironment; (**B**) immune infiltration; (**C**) immune checkpoint molecules; (**D**) immune cells. *P < 0.05, **P < 0.01, ***P < 0.001

In exploring the immune relevance of ADH5, it was discovered that ADH5 has a significant correlation with the immune score (*P* = 0.003, Fig. 7A), but it does not correlate with ESTIMATE and stromal scores. In terms of tumor immune cell infiltration, ADH5 was significantly associated with B-cell, CD8 + T-cell, and macrophage infiltration (all *P* < 0.001, Fig. 7B). Moreover, based on data from TCGA, we discovered that ADH5 is associated with immune checkpoint molecules, including CD200, CD40, HAVCR2, and HHLA2 in KIRC (all *P* < 0.05; Fig. 7C). Co-expression analysis of ADH5 and immune cell pathways showed that ADH5 was associated with activated B cell pathway, activated CD4 T cell pathway, activated CD8 T cell pathway, activated dendritic cell pathway, CD56dim natural killer cell pathway, central memory CD4 T cell pathway, macrophage pathway, MDSC pathway, Type 1 helper T cell pathway and Type 17 helper T cell pathway was negatively correlated. ADH5 was positively correlated with the immature dendritic cell pathway, Mast cell pathway, memory B cell pathway, and neutrophil pathway (all *P* < 0.05; Fig. 7D). In addition, Fig. 7A uses the ESTIMATE algorithm, a computerized algorithm that can be used to infer the level of stromal and immune-cell infiltration in tumor tissue based on expression profiling. The TIMER algorithm is used in Fig. 7B (the TIMER web server is a comprehensive resource for systematic analysis of immune infiltration in different cancer types. The abundance of multiple immune infiltrating cells was determined using the TIMER algorithm). The ssGSEA algorithm is used in Fig. 7D (the ssGSEA method is a recently proposed algorithm for counting immune cell subsets using RNA samples from various tissue types, including solid tumors). Differences in the different algorithms between them may lead to different correlations between ADH5 and different immune cells. Despite the differences in these algorithms, they all found a correlation between ADH5 and immunity.

### Immunotherapy reactivity associated with ADH5

**Fig. 8:**
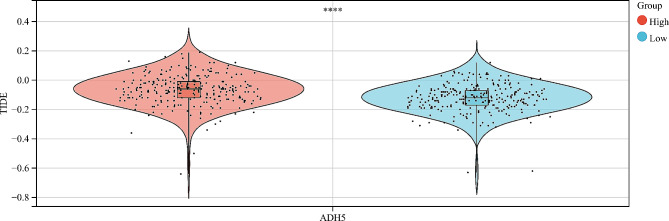
Forecasting the immune response to immunotherapy associated with ADH5

Our analysis of the TIDE database indicates that KIRC patients with high ADH5 expression have higher tumor immune dysfunction scores. This suggests that these KIRC patients with high ADH5 expression may not respond as well to immunotherapy as those with low ADH5 expression and may have a less favorable prognosis (Fig. 8).

### Single-cell analysis reveals ADH5 expression in immune cells

**Fig. 9:**
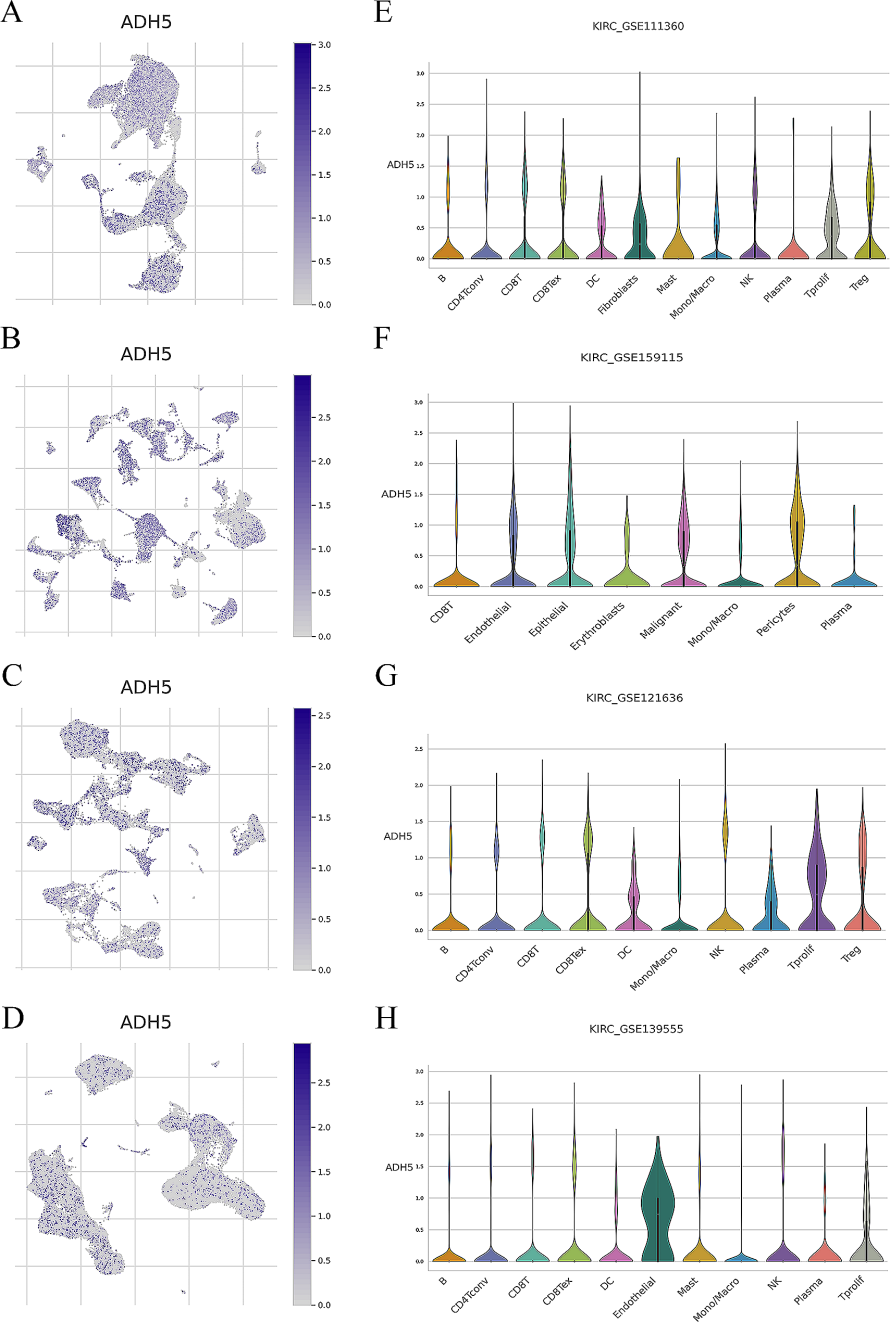
Single-cell analysis results. (**A**-**D**) Speckle plots showing ADH5 expression in each cell cluster in the four datasets, where the color depth is proportional to the expression level of the gene within the cell. (**E**-**H**) Violin plots showing the expression of ADH5 in different cell clusters in the four datasets

The UMAP plots for each cluster of the four datasets (GSE111360, GSE159115, GSE121636, and GSE139555) were visualized and labeled according to cell type. Different colors were used to represent different clusters of cells (Fig. S1 A-D). The speckle plots show the expression of ADH5 in each cell cluster, with darker colors meaning that the gene is more highly expressed in the cells. As displayed in Fig. 9A-D, ADH5 is expressed in tumor tissues and immune cells. The violin plot more visually demonstrates the expression of ADH5 in each cell cluster (Fig. 9E-H). Combined with the data of four groups of immune cells, we found that ADH5 was expressed in immune cells, and it may exert its immune effect through immune cells. These results partially explain the significant correlation between ADH5 and immunity at the single-cell level.

## Discussion

The incidence of renal cell carcinoma has been increasing over the past several decades, and its most common histologic subtype is clear cell carcinoma [19]. Finding new useful biomarkers to direct individualized treatment of KIRC and predict patient prognosis is crucial due to the high mortality rate that KIRC exhibits. Previous studies have shown that ADH5 is highly expressed in the liver, upper gastrointestinal tract, and kidney, and it is primarily involved in the metabolism of alcohols and aldehydes in humans [20]. Currently, in the field of oncology, there are also numerous studies showing a close association between ADH5 and cancer. Cañas et al. showed that increased ADH5 expression in HER2 breast cancer is associated with higher patient survival [21]. Besides, Chiang et al. found that dysregulation of ADH may exert a pathological contribution to the pathogenesis of rectal cancer [22]. However, to the best of our knowledge, none of the researchers have carried out an in-depth study on the role of ADH5 in clear cell renal cell carcinoma. Therefore, we have used bioinformatics to investigate the function of ADH5 in KIRC.

The study found that ADH5 was under-expressed in various tumors, including renal clear cell carcinoma, through pan-cancer profiling. Immunohistochemical results from the HPA database on ADH5 expression in KIRC and the CPTAC dataset on ADH5 expression in normal and KIRC tumor tissues were consistent with these results. This suggests that ADH5 may be an anti-oncogene. We further performed PCR to verify that ADH5 was also lowly expressed at the mRNA level in some KIRC cell types. Survival analysis showed that lower levels of ADH5 expression implied shorter survival times compared to the high-expression group. The correlation with clinicopathological analysis revealed that there was a positive correlation between ADH5 expression and the histological grade, clinical stage, and TMN stage of the patients (*p* < 0.05). In addition, ADH5 was identified as a separate factor influencing the prognosis of KIRC patients by univariate and multifactorial Cox regression.

Immediately afterward, we applied GSEA to identify the signaling pathways associated with ADH5. The results showed that ADH5 is mainly involved in a variety of substrate metabolism processes, in cell growth processes, and in the composition of the immune microenvironment. The increased incidence of human hepatocellular carcinoma (HCC) was demonstrated by Tang et al. to be related to reduced activity of the ADH5-mediated DNA repair protein O6-alkylguanine-DNA alkyltransferase [23]. Among the five signaling pathways obtained, the mTOR signaling pathway is frequently activated, and regulation of mTOR activity is frequently lost in various human cancers, such as breast, prostate, lung, liver, and kidney cancers [24]. The most classical of these signaling pathways belongs to the TGF-β signaling pathway. The pathogenesis of fibrosis and cancer is underpinned by defects in the TGF-β signaling pathway [25]. In the early stages of cancer, TGF-β inhibits tumor growth by suppressing cell cycle progression and promoting apoptosis, resulting in cell cycle arrest and apoptosis. However, in advanced stages, TGF-β has a tumor-promoting effect, increasing tumor aggressiveness and metastasis [26].

We constructed a PPI network and performed association screens for a total of 10 genes to understand the potential interactions of ADH5 with other genes. In addition, we carried out a correlation analysis to explore the possibility of ADH5 in KIRC immunotherapy, which found no clear association between ADH5 and MSI, TMB, and TNB.

The tumor immune microenvironment plays an important role in tumor diagnosis, prevention, treatment, and prognosis [27]. Immune cells infiltrating tumors are an essential component of the tumor microenvironment, which is intimately linked to tumor cell proliferation, treatment response, and prognosis [28]. Whereas RCC has always been a cancer with high levels of immune cell infiltration [29]. The analysis of KIRC immune infiltration in this study revealed a significant association between ADH5 and B-cell, CD8 + T-cell, and macrophage infiltration. B-cell infiltration has been reported to prolong cancer-specific survival [30]. As the effector cells of tumor immunotherapy, CD8 + T cells help renal cell carcinoma spread by blocking the DAB2IP signaling pathway, which is a tumor suppressor gene [31]. The mechanism may be through increasing the expression of estrogen receptor β (ERβ) in renal cell carcinoma, and the expression of ERβ is closely related to the clinical prognosis and immunotherapy response of renal cell carcinoma [32]. In addition, according to Chuanjie Zhang et al., macrophage levels were negatively correlated with KIRC prognosis [33].

Based on data from TCGA, we discovered a correlation between ADH5 and immune checkpoint molecules, including CD200, CD40, HAVCR2, and HHLA2 in KIRC. Immune checkpoint molecules are distinct surface proteins that activated lymphocytes express to control the immune response [34]. They control the immune response and keep things in balance by interacting with ligands and receptors [35]. However, if tumor cells express tumor antigens capable of evading the immune system, the balance is altered, and cancer progression becomes apparent due to dynamic and uncontrolled cell growth [36, 37]. The composition of the tumor microenvironment (TME) has proven to impact the effect of the immune checkpoint blockade (ICB) response on tumors [38].

This study used the Tumor Immune Dysfunction and Exclusion (TIDE) algorithm to predict clinical response to immune checkpoint inhibitors. The TIDE algorithm is a computational method that models the two main mechanisms of tumor immune escape, with higher tumor TIDE prediction scores indicating poorer treatment efficacy with immune checkpoint inhibitors [39]. As a result, the group with low expression of ADH5 demonstrated a greater response to immunotherapy. Finally, advances in single-cell RNA sequencing (scRNA-seq) enable us to analyze immune system patterns and study mechanisms of tumor progression to some extent [40]. Single-cell analysis downloaded from the GEO database and analyzed showed that the ADH5 gene can be expressed in immune cells, suggesting that it can act by regulating immune cells. This finding partially supports the possibility that ADH5 can predict immune responses.

Overall, this study has several implications. Our results suggest that ADH5 is an anti-oncogene. It is a biomarker of response prognosis and a potential therapeutic target for KIRC patients. In addition, ADH5 may be a predictor of response to immunotherapy and is therefore worthy of further investigation. However, there are still some shortcomings in this paper. On the one hand, we did not experimentally validate ADH5 expression in terms of protein level; therefore, its expression still lacks valid and accurate verification. On the other hand, the specific mechanisms associated with ADH5 affecting KIRC in this paper still need further investigation.

## Conclusion

In conclusion, this paper has validated the differences in ADH5 expression between KIRC tissues and normal tissues by means of bioinformatic analysis and has demonstrated its potential as a prognostic biomarker and as a therapeutic target for KIRC. Furthermore, the expression of ADH5 in KIRC was found to correlate with the level of immune cell infiltration, suggesting that ADH5 is probably important for the regulation of the immune microenvironment in KIRC. The TIDE algorithm also showed that patients who have low ADH5 expression respond better to immunotherapy. However, we still need more research to support our findings.

### Electronic supplementary material

Below is the link to the electronic supplementary material.

Supplementary Material 1

## Data Availability

The RNA-sequencing data and corresponding clinical information were downloaded from the Cancer Genome Atlas (TCGA) database (https://portal.gdc.cancer.gov/).

## References

[1] Yildirim C, Kutluhan MA, Sahin A, Topakta R, Urkmez A (2020). Three different urogenital carcinoma in an aging patient: a rare case presentation. Aging Male.

[2] John A, Spain L, Hamid AA (2023). Navigating the current Landscape of Non-clear Cell Renal Cell Carcinoma: a review of the literature. Curr Oncol.

[3] Meng J, Gao L, Zhang M, Gao S, Fan S, Liang C (2020). Systematic investigation of the prognostic value of cell division cycle-associated proteins for clear cell renal cell carcinoma patients. Biomark Med.

[4] You B, Sun Y, Luo J, Wang K, Liu Q, Fang R, Liu B, Chou F, Wang R, Meng J (2021). Androgen receptor promotes renal cell carcinoma (RCC) vasculogenic mimicry (VM) via altering TWIST1 nonsense-mediated decay through lncRNA-TANAR. Oncogene.

[5] Bian Z, Meng J, Niu Q, Jin X, Wang J, Feng X, Che H, Zhou J, Zhang L, Zhang M (2020). Prognostic role of Prothrombin Time Activity, Prothrombin Time, Albumin/Globulin ratio, platelets, sex, and Fibrinogen in Predicting recurrence-free Survival Time of Renal Cancer. Cancer Manag Res.

[6] Chida K, Oshi M, Roy AM, Sato T, Endo I, Takabe K (2023). Pancreatic ductal adenocarcinoma with a high expression of alcohol dehydrogenase 1B is associated with less aggressive features and a favorable prognosis. Am J Cancer Res.

[7] Lee DW, Ji YB, Song CM, Kim JK, Lee SH, Tae K. Impact of Alcohol dehydrogenase 7 polymorphism and alcohol consumption on risk of Head and Neck squamous cell carcinoma: a Korean case-control study. J Clin Med 2023, 12(14). 10.3390/jcm12144653 PMC10380624 37510768

[8] Du Q, Liu S, Dong K, Cui X, Luo J, Geller DA (2023). Downregulation of iNOS/NO promotes epithelial-mesenchymal transition and metastasis in Colorectal Cancer. Mol Cancer Res.

[9] Kasamatsu S, Nishimura A, Alam MM, Morita M, Shimoda K, Matsunaga T, Jung M, Ogata S, Barayeu U, Ida T (2023). Supersulfide catalysis for nitric oxide and aldehyde metabolism. Sci Adv.

[10] Cañas A, López-Sánchez LM, Peñarando J, Valverde A, Conde F, Hernández V, Fuentes E, López-Pedrera C, de la Haba-Rodríguez JR, Aranda E (2016). Altered S-nitrosothiol homeostasis provides a survival advantage to breast cancer cells in HER2 tumors and reduces their sensitivity to trastuzumab. Biochim Biophys Acta.

[11] Al-Refai R, Bendari A, Morrar D, Sham S, Kataw L, Garajayev A, Hajiyeva S. Immunohistochemical Staining Characteristics of Low-Grade Invasive Ductal Carcinoma Using the ADH5 Cocktail (CK5/14, P63, and CK7/18): A Potential Interpretative Pitfall. *Diagnostics (Basel)* 2023, 13(18). 10.3390/diagnostics13182966 PMC10527570 37761331

[12] Zhang Y, Zhang R, Liang F, Zhang L, Liang X (2020). Identification of Metabolism-Associated prostate Cancer subtypes and Construction of a prognostic risk model. Front Oncol.

[13] Kanehisa M, Furumichi M, Sato Y, Kawashima M, Ishiguro-Watanabe M (2023). KEGG for taxonomy-based analysis of pathways and genomes. Nucleic Acids Res.

[14] Wang Y, Cong R, Liu S, Zhu B, Wang X, Xing Q (2021). Decreased expression of METTL14 predicts poor prognosis and construction of a prognostic signature for clear cell renal cell carcinoma. Cancer Cell Int.

[15] Luo Y, Shen D, Chen L, Wang G, Liu X, Qian K, Xiao Y, Wang X, Ju L (2019). Identification of 9 key genes and small molecule drugs in clear cell renal cell carcinoma. Aging.

[16] Guo Y, Wu Z, Cen K, Bai Y, Dai Y, Mai Y, Hong K, Qu L (2023). Establishment and validation of a ubiquitination-related gene signature associated with prognosis in pancreatic duct adenocarcinoma. Front Immunol.

[17] Zhai W, Chen S, Duan F, Wang J, Zhao Z, Lin Y, Rao B, Wang Y, Zheng L, Long H. Risk stratification and prognosis prediction based on inflammation-related gene signature in lung squamous carcinoma. Cancer Med 2022. 10.1002/cam4.5190 PMC9972108 36056909

[18] Szklarczyk D, Kirsch R, Koutrouli M, Nastou K, Mehryary F, Hachilif R, Gable AL, Fang T, Doncheva NT, Pyysalo S (2023). The STRING database in 2023: protein-protein association networks and functional enrichment analyses for any sequenced genome of interest. Nucleic Acids Res.

[19] Yu Z, Lv Y, Su C, Lu W, Zhang R, Li J, Guo B, Yan H, Liu D, Yang Z (2023). Integrative Single-Cell Analysis Reveals Transcriptional and Epigenetic Regulatory Features of Clear Cell Renal Cell Carcinoma. Cancer Res.

[20] Feurstein S (2023). Emerging bone marrow failure syndromes- new pieces to an unsolved puzzle. Front Oncol.

[21] Liu Q, Gu T, Su LY, Jiao L, Qiao X, Xu M, Xie T, Yang LX, Yu D, Xu L (2021). GSNOR facilitates antiviral innate immunity by restricting TBK1 cysteine S-nitrosation. Redox Biol.

[22] Chiang CP, Jao SW, Lee SP, Chen PC, Chung CC, Lee SL, Nieh S, Yin SJ (2012). Expression pattern, ethanol-metabolizing activities, and cellular localization of alcohol and aldehyde dehydrogenases in human large bowel: association of the functional polymorphisms of ADH and ALDH genes with hemorrhoids and colorectal cancer. Alcohol.

[23] Tang CH, Wei W, Liu L (2012). Regulation of DNA repair by S-nitrosylation. Biochim Biophys Acta.

[24] Glaviano A, Foo ASC, Lam HY, Yap KCH, Jacot W, Jones RH, Eng H, Nair MG, Makvandi P, Geoerger B (2023). PI3K/AKT/mTOR signaling transduction pathway and targeted therapies in cancer. Mol Cancer.

[25] Alam J, Huda MN, Tackett AJ, Miah S (2023). Oncogenic signaling-mediated regulation of chromatin during tumorigenesis. Cancer Metastasis Rev.

[26] Massagué J, Sheppard D (2023). TGF-β signaling in health and disease. Cell.

[27] Ke J, Chen J, Liu X (2022). Analyzing and validating the prognostic value and immune microenvironment of clear cell renal cell carcinoma. Anim Cells Syst (Seoul).

[28] Cao H, Zhang J, Wang W (2020). DAB2IP plays important clinical significance and correlates with Immune Infiltration in Renal Cell Carcinoma. Technol Cancer Res Treat.

[29] Krishna C, DiNatale RG, Kuo F, Srivastava RM, Vuong L, Chowell D, Gupta S, Vanderbilt C, Purohit TA, Liu M (2021). Single-cell sequencing links multiregional immune landscapes and tissue-resident T cells in ccRCC to tumor topology and therapy efficacy. Cancer Cell.

[30] Stenzel PJ, Schindeldecker M, Tagscherer KE, Foersch S, Herpel E, Hohenfellner M, Hatiboglu G, Alt J, Thomas C, Haferkamp A (2020). Prognostic and predictive value of Tumor-infiltrating leukocytes and of Immune Checkpoint molecules PD1 and PDL1 in Clear Cell Renal Cell Carcinoma. Transl Oncol.

[31] Yeh CR, Ou ZY, Xiao GQ, Guancial E, Yeh S (2015). Infiltrating T cells promote renal cell carcinoma (RCC) progression via altering the estrogen receptor β-DAB2IP signals. Oncotarget.

[32] Ou Z, Wang Y, Chen J, Tao L, Zuo L, Sahasrabudhe D, Joseph J, Wang L, Yeh S (2018). Estrogen receptor β promotes bladder cancer growth and invasion via alteration of miR-92a/DAB2IP signals. Exp Mol Med.

[33] Zhang C, Li Z, Qi F, Hu X, Luo J (2019). Exploration of the relationships between tumor mutation burden with immune infiltrates in clear cell renal cell carcinoma. Ann Transl Med.

[34] Kanannejad Z, Soleimanian S, Ghahramani Z, Sepahi N, Mohkam M, Alyasin S, Kheshtchin N (2023). Immune checkpoint molecules in prevention and development of asthma. Front Immunol.

[35] Moon SY, Han M, Ryu G, Shin SA, Lee JH, Lee CS. Emerging Immune Checkpoint molecules on Cancer cells: CD24 and CD200. Int J Mol Sci 2023, 24(20). 10.3390/ijms242015072 PMC10606340 37894750

[36] Mao X, Xu J, Wang W, Liang C, Hua J, Liu J, Zhang B, Meng Q, Yu X, Shi S (2021). Crosstalk between cancer-associated fibroblasts and immune cells in the tumor microenvironment: new findings and future perspectives. Mol Cancer.

[37] De Felice F, Musio D, Tombolini V. Immune check-point inhibitors and Standard Chemoradiotherapy in definitive Head and Neck Cancer Treatment. J Pers Med 2021, 11(5). 10.3390/jpm11050393 PMC8151395 34068797

[38] Petitprez F, Meylan M, de Reyniès A, Sautès-Fridman C, Fridman WH (2020). The Tumor Microenvironment in the response to Immune Checkpoint Blockade therapies. Front Immunol.

[39] Li X, Gao Z, Chen J, Feng S, Luo X, Shi Y, Tang Z, Liu W, Zhang X, Huang A (2023). Integrated single cell and bulk sequencing analysis identifies tumor reactive CXCR6(+) CD8 T cells as a predictor of immune infiltration and immunotherapy outcomes in hepatocellular carcinoma. Front Oncol.

[40] Camps J, Noël F, Liechti R, Massenet-Regad L, Rigade S, Götz L, Hoffmann C, Amblard E, Saichi M, Ibrahim MM (2023). Meta-analysis of Human Cancer single-cell RNA-Seq datasets using the IMMUcan Database. Cancer Res.

