# Supplementary material for: Elevated ADH5 expression suggested better prognosis in kidney renal clear cell carcinoma (KIRC) and related to immunity through single-cell and bulk RNA-sequencing
Source: BMC Urol. 2024 Apr 10;24:84. doi: 10.1186/s12894-024-01478-9 (PMC11007970; doi:10.1186/s12894-024-01478-9)

**Fig.S1**. Single-cell analysis results. UMAP plots of the (A) GSE111360, (B) GSE159115, (C) GSE121636, and (D) GSE139555 data sets, and each cluster was visualized and labeled.


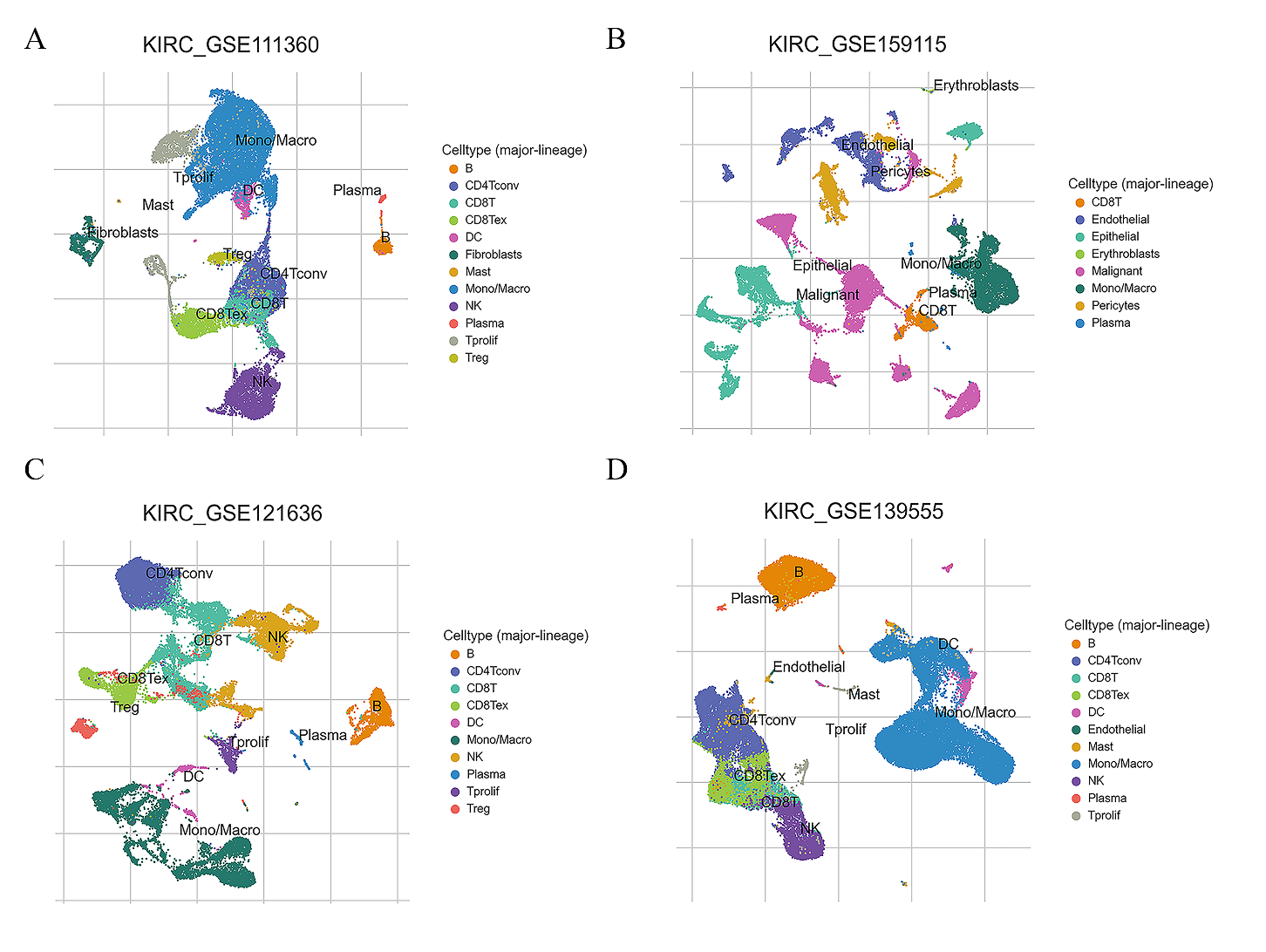

Supplement: Supplementary file 1 — Supplementary Material 1 [file 12894_2024_1478_MOESM1_ESM.docx]
